# Dose‐Escalated Upadacitinib for Refractory Myositis in Anti‐Jo‐1‐Positive Antisynthetase Syndrome: A Case Report

**DOI:** 10.1155/crrh/5972090

**Published:** 2026-09-17

**Authors:** Yuji Kukida, Hironori Inoue

**Affiliations:** ^1^ Department of Rheumatology, Japanese Red Cross Kyoto Daini Hospital, Kyoto, Japan, jrc.or.jp; ^2^ Inflammation and Immunology, Graduate School of Medical Science, Kyoto Prefectural University of Medicine, Kyoto, Japan, kpu-m.ac.jp

**Keywords:** anti-jo-1 antibody, antisynthetase syndrome, case report, JAK inhibitor, upadacitinib

## Abstract

Anti‐Jo‐1‐positive antisynthetase syndrome (ASS) can follow a relapsing course during glucocorticoid tapering, and evidence for refractory myositis remains limited. We report a 33‐year‐old woman with anti‐Jo‐1‐positive ASS with a dermatomyositis phenotype and recurrent myositis despite multiple immunosuppressants, biologics, and Janus kinase (JAK) inhibitors. Upadacitinib was increased from 15 to 30 mg/day during a flare, together with intravenous immunoglobulin and temporary prednisolone escalation. During follow‐up, upadacitinib was withdrawn twice for fertility treatment; both withdrawals were followed by disease flares, and disease control was restored after reintroduction. Prednisolone was ultimately discontinued. This repeated withdrawal‐rechallenge pattern suggests that dose‐escalated upadacitinib may have contributed to sustained disease control.

## 1. Introduction

Antisynthetase syndrome (ASS) is an idiopathic inflammatory myopathy characterized by myositis, arthritis, interstitial lung disease (ILD), and other systemic manifestations in association with anti‐aminoacyl‐tRNA synthetase antibodies. Recurrent myositis can hinder glucocorticoid tapering, and treatment options for refractory disease remain limited [1]. Janus kinase (JAK) inhibitors have shown potential benefit in refractory inflammatory myopathies [2–9], but experience with upadacitinib remains limited [7]. We report a highly relapsing case of anti‐Jo‐1‐positive ASS in which sustained disease control was observed during treatment with dose‐escalated upadacitinib.

## 2. Case Presentation

A 33‐year‐old woman was referred with myalgia and proximal muscle weakness involving the bilateral deltoid, iliopsoas, and quadriceps muscles. Physical examination also showed mild heliotrope rash and Gottron’s sign, clinically apparent polyarthritis involving the proximal interphalangeal, metacarpophalangeal, ankle, and metatarsophalangeal joints, and Raynaud’s phenomenon. Mechanic’s hands and dysphagia were absent. Serum creatine kinase (CK) was 8597 U/L (reference range, 41–153 U/L), and C‐reactive protein (CRP) was 0.49 mg/dL (reference range, < 0.14 mg/dL). Anti‐Jo‐1 antibody was > 500 U/mL (reference range, < 10.0 U/mL) by chemiluminescent enzyme immunoassay; 500 U/mL was the upper limit of quantification. Anti‐SS‐A/Ro antibody was also positive. Chest computed tomography showed minimal bilateral lower lobe subpleural ground‐glass and reticular opacities consistent with a nonspecific interstitial pneumonia pattern. She had no respiratory symptoms, and pulmonary function testing was not performed. She was diagnosed with anti‐Jo‐1‐positive ASS with a dermatomyositis phenotype.

Treatment was started with prednisolone (PSL) 60 mg/day and tacrolimus 2‐3 mg/day. Cyclophosphamide was avoided because of concern about gonadotoxicity in a patient wishing to preserve fertility. CK decreased to 55 U/L, and CRP to < 0.09 mg/dL (assay detection limit, 0.09 mg/dL), and follow‐up chest computed tomography at 1 year showed resolution of the ILD. The skin lesions did not recur. During PSL tapering, however, repeated flares occurred with myalgia, CK elevation (approximately 200–1250 U/L), CRP elevation (1–5 mg/dL), and occasional knee arthralgia, without recurrent objective muscle weakness, skin disease, or ILD. For this report, a flare was defined as recurrence or worsening of myalgia accompanied by a concurrent CK increase, with or without arthralgia, prompting treatment intensification.

Several glucocorticoid‐sparing therapies failed to maintain remission: tacrolimus 2‐3 mg/day for 25 months; methotrexate 6 mg/week for 1 month, discontinued because of primary treatment failure; mycophenolate mofetil 1000–2500 mg/day for 9 months; rituximab 375 mg/m^2^ weekly for 4 weeks; tofacitinib 10 mg/day for 5 months; abatacept 500 mg intravenously at weeks 0, 2, and 4; baricitinib 4 mg/day for 9 months; and upadacitinib 15 mg/day for 3 months.

**FIGURE 1:**
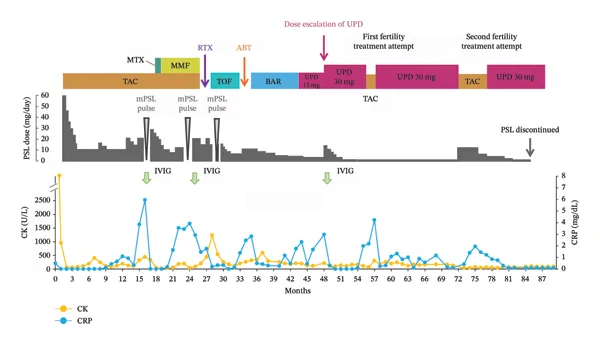
Clinical course of the case at approximately Month 48, upadacitinib was increased from 15 to 30 mg/day concurrently with an increase in PSL from 5 to 20 mg/day and IVIG 20 g/day for 5 consecutive days. Upadacitinib was temporarily discontinued twice for fertility treatment; both withdrawals were followed by disease flares, and treatment was resumed. PSL was discontinued at Month 86. TAC, tacrolimus; MTX, methotrexate; MMF, mycophenolate mofetil; RTX, rituximab; ABT, abatacept; TOF, tofacitinib; BAR, baricitinib; UPD, upadacitinib; PSL, prednisolone; mPSL, methylprednisolone; IVIG, intravenous immunoglobulin; CK, creatine kinase; CRP, C‐reactive protein.

At Month 48, while receiving PSL 5 mg/day and upadacitinib 15 mg/day, thigh myalgia recurred with CK 215 U/L and CRP 2.97 mg/dL. There was no objective muscle weakness, recurrent skin disease, or ILD activity. Upadacitinib was increased to 30 mg/day concurrently with PSL escalation to 20 mg/day and intravenous immunoglobulin 20 g/day for 5 days. No other immunosuppressive therapy was changed. Thereafter, disease activity remained controlled on upadacitinib 30 mg/day without further intravenous immunoglobulin or glucocorticoid escalation (Figure 1).

Upadacitinib was subsequently interrupted twice to permit fertility treatment. Each interruption was followed by a disease flare, requiring reintroduction of upadacitinib. After the second flare, the patient decided not to pursue further pregnancy attempts; upadacitinib was continued, and PSL was ultimately discontinued at Month 86. She had been counseled to avoid pregnancy during JAK‐inhibitor therapy.

Pretreatment screening for tuberculosis and viral hepatitis was negative, routine laboratory monitoring showed no clinically significant abnormalities, and recombinant zoster vaccination was administered during treatment. No serious infections, herpes zoster, or cytomegalovirus reactivation occurred during treatment; only mild nasopharyngitis and herpes labialis were observed.

## 3. Discussion

This case illustrates a highly relapsing course of anti‐Jo‐1‐positive ASS with a dermatomyositis phenotype, in which muscle activity evolved independently of the cutaneous and pulmonary manifestations. Anti‐Jo‐1‐positive ASS is associated with recurrent myositis [1, 10, 11], and a recent study of 115 patients showed that longitudinal changes in anti‐Jo‐1 antibody levels were associated with changes in global, muscle, and pulmonary disease activity [12]. Anti‐SS‐A/Ro antibody positivity has also been associated with relapse in IIM [13]. The strongly positive anti‐Jo‐1 antibody and concomitant anti‐SS‐A/Ro positivity were consistent with the highly relapsing disease course observed in this patient.

Type I interferon (IFN) signaling is an established pathogenic pathway in IIM, and type II IFN signaling is also activated in ASS [14–21]. JAK inhibitors can modulate both pathways and have shown potential efficacy in refractory IIM [6, 8, 9]. A previous case series reported improvement in cutaneous disease activity with upadacitinib in refractory IIM, including ASS, after incomplete response to tofacitinib [7]. Traves et al. demonstrated that pharmacodynamic inhibition of cytokine signaling, including IFN‐γ‐induced pSTAT1, differs among JAK inhibitors at clinically relevant exposures and that higher upadacitinib exposure can increase predicted pathway inhibition [22]. These findings indicate that nominal JAK selectivity alone does not predict pharmacodynamic effects; however, they do not explain why this patient achieved more sustained disease control with upadacitinib 30 mg/day than with tofacitinib, baricitinib, or upadacitinib 15 mg/day.

Clinical observations nevertheless suggest that dose escalation may improve efficacy in selected refractory settings. In anti‐MDA5 antibody‐positive dermatomyositis, escalation of tofacitinib from 10 to 20 mg/day has been reported to control refractory disease after an inadequate response to 10 mg/day [23, 24]. Moreover, Phase 3 trials of upadacitinib in atopic dermatitis and as maintenance therapy for ulcerative colitis and Crohn’s disease showed generally higher efficacy outcomes with 30 mg/day than with 15 mg/day [25–27]. These findings suggest that increasing JAK‐inhibitor exposure may be a potential strategy in selected treatment‐refractory cases. However, they do not establish that the response in this patient was dose dependent, and the efficacy and safety of JAK‐inhibitor dose escalation in IIM remain uncertain.

A central limitation in interpreting the effect of upadacitinib dose escalation is that IVIG and a temporary increase in PSL were administered concurrently at the 48‐month flare. Accordingly, the short‐term response cannot be attributed to upadacitinib alone. During subsequent follow‐up, upadacitinib was withdrawn twice for fertility treatment; both withdrawals were followed by disease flares, and disease control was restored after reintroduction without further IVIG or glucocorticoid dose escalation. After the patient decided not to pursue further pregnancy attempts, treatment was continued and PSL was ultimately discontinued. This repeated withdrawal‐rechallenge pattern supports a possible contribution of upadacitinib to sustained disease control, although it does not establish causality in an uncontrolled single‐patient observation.

Safety is particularly relevant when considering prolonged off‐label treatment with upadacitinib 30 mg/day. Higher JAK‐inhibitor exposure may increase the risk of adverse events, including infections, and careful screening and longitudinal monitoring are required. In this patient, tuberculosis and viral hepatitis screening were performed, hematologic, hepatic, and lipid parameters were monitored, recombinant zoster vaccination was administered, and no serious infectious adverse event was observed. Nevertheless, the safety of this strategy cannot be inferred from a single case. Complete standardized longitudinal MMT‐8 assessments were not available, which limits the assessment of longitudinal muscle strength.

In summary, the sustained disease control and repeated withdrawal‐rechallenge pattern observed in this case suggest that dose‐escalated upadacitinib may warrant further investigation as a potential therapeutic strategy for selected patients with refractory anti‐Jo‐1‐positive ASS.

## Funding

This report received no specific grant from any funding agency.

## Ethics Statement

Ethical approval was not required for this case report in accordance with institutional policy.

## Consent

Written informed consent for publication was obtained from the patient.

## Conflicts of Interest

Yuji Kukida received speaker fees from AbbVie. AbbVie had no role in medication access or provision, treatment decisions, data collection, data analysis or interpretation, medical‐writing assistance, manuscript preparation, or the decision to submit the manuscript.

## Data Availability

Data are available from the corresponding author upon reasonable request.
